# Colorectal Cancer Research: Basic, Preclinical, and Clinical Approaches

**DOI:** 10.3390/cancers12020416

**Published:** 2020-02-11

**Authors:** Jean-François Beaulieu

**Affiliations:** 1 Laboratory of Intestinal Physiopathology, Faculty of Medicine and Health Sciences, Université de Sherbrooke, Sherbrooke, QC J1H 5N4, Canada; Jean-Francois.Beaulieu@USherbrooke.ca; Tel.: +1-819-821-8000 (ext. 75269); 2 Centre de Recherche du Centre Hospitalier Universitaire de Sherbrooke, Sherbrooke, QC J1H 5N4, Canada

Colorectal cancer remains one of the deadliest cancers worldwide. It has become evident that further efforts in colorectal cancer research are required, from providing a better understanding of the cellular and molecular mechanisms leading to colorectal neoplasm initiation and progression from adenoma to metastasis, to generating reliable non-invasive detection tests for identifying lesions at early stages, as well as refining the current therapeutic and personalized approaches and developing new ones. The aim of this Special Issue is to cover all aspects of colorectal cancer research, including basic, preclinical, and clinical approaches.

The original articles of this Special Issue present innovative findings toward the design of new strategies that may contribute to the fight against colorectal cancer cells, such as treatments that enhance apoptosis-related mechanisms [1,2], chemosensitivity [3,4,5,6] and/or radiotherapy [7], and the characterization of new pathways that lead to the identification of specific biomarkers and/or decipher unique mechanisms underlying colorectal cancer initiation and progression. These mechanisms include the transcriptional regulation of DNA repair proteins [8]; the epigenetic regulation of the zinc finger E-box-binding homeobox 1 [9] and the expression of the (pro)renin receptor [10], WNT-11 [11], and calcium and calcium-activated potassium channels [12]; the contribution of immune cells in the tumor microenvironment [13]; and the identification of genetic aberrations that occur during the transition from adenoma to carcinoma [14]. The development of tools for preclinical research is also a key research area, such as the establishment of tumor models at the individual patient level [15] and the refinement of clinical approaches for improving the survival of patients with metastatic colorectal cancer [16,17,18,19].

Review articles have summarized the latest developments of clinical issues related to the management of improving chemotherapeutic approaches for metastatic colorectal cancer [20,21,22], the characterization of the hallmarks of serrated colorectal lesions [23] and immune-mediated intestinal disorders that can be associated with small bowel carcinoma [24], as well as the current state of knowledge of specific facets of colorectal cancer research such as oncogenic tyrosine kinase signaling [25], integrin α6β4 [26], and epigenetic mechanisms by which long non-coding RNAs regulate gene expression [27]. 

The variety of the content of this Special Issue devoted to colorectal cancer illustrates the need for investigating the cellular basis of this disease from every angle in order to develop efficient screening and treatment strategies that can significantly impact the quality of life of the patients. I would like to thank all of the authors that have contributed to the articles of this Special Issue for their work.
